# Assessing the capacity to diagnose human African trypanosomiasis among health care personnel from Chama and Mambwe districts of eastern Zambia

**DOI:** 10.1186/s13104-015-1403-6

**Published:** 2015-09-11

**Authors:** Gloria M. Mulenga, Rosemary N. Likwa, Boniface Namangala

**Affiliations:** Department of Public Health, School of Medicine, University of Zambia, Lusaka, Zambia; Institute of Distance Education, The University of Zambia, Lusaka, Zambia

**Keywords:** Human African trypanosomiasis, Diagnosis, Health care personnel, Rural health centres, Zambia

## Abstract

**Background:**

Human African Trypanosomiasis (HAT) is a neglected tropical disease affecting poor rural communities living in tsetse-infested regions of sub-Saharan Africa. In Zambia, sporadic cases of HAT have been reported mainly in the old foci along the tsetse-infested Luangwa river valley in north-eastern part of the country. In such places where malaria is the major endemic febrile disease, with possibilities of co-infections of HAT and malaria and where the levels of alertness to the presence of HAT among health care personnel (HCP) is low, there is a high chance of misdiagnosing HAT for malaria because of their similarities in clinical presentation. This study, conducted in Zambia’s tsetse-infested rural health centres (RHCs) of Chama and Mambwe districts, was designed to investigate the staffing levels, the HCP levels of alertness to the occurrence of HAT and their capacity to detect the disease.

**Methods:**

Structured questionnaires were used to collect information pertaining to HAT alertness and the capacity to detect the disease from 101 HCP in a cross sectional study of 23 RHCs drawn from Zambia’s Chama and Mambwe districts between April and July 2013. The data collected were analyzed using Stata/SE version 11.0.

**Results:**

Participants from both Chama and Mambwe district RHCs reported similar very low levels of qualified HCP and laboratory technicians, and that they had similar basic tools for HAT diagnosis. Although not statistically significant, respondents from Chama (~89 %) tended to be more aware about the occurrence of HAT compared to their Mambwe counterparts (~78 %). Whereas ~40 % of the HCP from Chama district (n = 52) claimed to have encountered at least one case of HAT, only ~4 % of their Mambwe counterparts (n = 49) had similar experiences (P = 0.000).

**Conclusion:**

Health care personnel in RHCs from Chama tended to be more alert to the occurrence of HAT than the HCP from Mambwe district. The extremely low levels of categorized HCP, general absence of functional laboratories, coupled with absence of national HAT surveillance and control programs, are among some of the serious challenges that Zambia’s Chama and Mambwe districts face to control/eliminate HAT.

## Findings

### Background

Human African trypanosomiasis (HAT) is a tsetse-transmitted disease caused by *Trypanosoma brucei rhodesiense* (southern and eastern Africa) and *T. b. gambiense* (west and central Africa) [1]. In Zambia, HAT is caused by *T. b. rhodesiense*, with many species of game and domestic animals harbouring the parasite and sustaining sporadic transmission to humans [2–5]. Encroachment of people and their livestock into tsetse-infested areas and the subsequent disappearance of large game animals because of human interference, force tsetse flies to feed on humans and their livestock [6]. Historically, epidemics of *rhodesiense* HAT were reported from the north and south Luangwa valley and the Kafue river valley in the 1960s and early 1970s [7]. According to world health organisation (WHO) [8], Zambia currently reports <100 new HAT cases annually, mainly from the old foci in the tsetse-infested Luangwa river valley in eastern and north-eastern parts of the country, including Chama [4] and Mpika [5] districts, where the disease is re-emerging [4]. Despite past tsetse control efforts in eastern Zambia, HAT has never been eliminated due to the absence of a national HAT and tsetse fly control programme [9]. Thus, HAT remains an important public health problem in eastern Zambia, particularly among the poor and marginalised people, living in remote rural areas where access to medical facilities is limited. Although treatable, several *rhodesiense* HAT cases in endemic areas such as eastern Zambia have continued to go undiagnosed due to lack of deliberate disease surveillance programmes and lack of functional laboratories in remote rural health centres (RHCs), leading to serious socio-economic consequences [1].

HAT comprises an early stage, characterized by the presence of parasites in blood and lymph and a late stage, characterized by parasite invasion of the central nervous system (CNS) [8, 10]. Symptoms of early stage HAT are non-specific and include headache, fever and nausea [10, 11]. In the late stage, the disease is characterized by severe neurological disorders such as behavioural changes, sleep disorders, severe wasting and organ malfunction that eventually leads to death [10, 11]. In endemic regions, *rhodesiense* HAT may be confused with other endemic febrile diseases such as malaria, enteric fever, tuberculosis, meningitis and HIV/AIDS due to their similar clinical signs [12]. Therefore, clinical suspicions of HAT must be confirmed by laboratory tests [13].

It is noteworthy that *T. b. gambiense* and *T. b. rhodesiense* are phenotypically identical by microscopy [13]. Although microscopy is associated with low sensitivity due to fluctuating parasitaemia in *gambiense* HAT patients, parasitological confirmation is relatively easier in *rhodesiense* HAT patients because bloodstream trypanosomes are numerous [13]. In such *rhodesiense* HAT patients, trypanosomes are sometimes detected by chance while searching for other haemoparasites such as *Plasmodium* spp (malaria parasites) [14]*. Trypanosoma b. rhodesiense* parasites may also be detected by microscopic examination of chancre aspirates shortly after the infective bite by a tsetse fly [13]. Although microscopy is the WHO standard reference for *rhodesiense* HAT detection in patients, this technique may give false negative results during the remission phase of parasitaemia [13]. However, molecular techniques such as polymerase chain reaction (PCR) and the Loop-mediated isothermal amplification (LAMP), although not yet validated by WHO for HAT diagnosis beyond research settings, have higher sensitivity and specificity for trypanosome detection [4, 13, 15]. Thus, LAMP and PCR may be used to determine infections when the parasitaemia is below the threshold of direct microscopic observation. Although HAT has been re-emerging in most of the old foci within sub-Saharan Africa since the 1970s, with *T. b. gambiense* accounting for more than 98 % of the reported cases [16], the latest WHO reports suggest that the number of new cases have reduced [8]. In 2009, the number of cases reported dropped below 10,000 (9878) for the first time in 50 years, which further dropped to 7216 in 2012 [8]. Continuous control efforts in affected areas have attributed to the decrease in the number of new cases reported [7]. However, despite such progress, only a fraction of the population at HAT risk in sub-Saharan Africa (SSA) is under surveillance and relatively few cases are diagnosed annually [1, 17]. In particular, there is considerable under-diagnosis of *rhodesiense* HAT in SSA, including Zambia, mainly due to lack of HAT surveillance and control programmes [14, 18, 19]. Translation of disease diagnostic knowledge into proper care of patients is among the critical areas in health care delivery. This is only possible if health service providers posses the right knowledge of the health problems they are dealing with [13]. It is assumed that the more years of health care service for the health staff in a particular district, the more knowledge and experience gained from the type and occurrences of diseases in the area. On the other hand, inadequate knowledge of any aspect of a disease is a potential contributing factor to disease misdiagnosis [20]. Therefore, investigating the levels of knowledge of HAT diagnosis among health personnel and the diagnostic capacity of health centres may be an important step towards identifying gaps in the current diagnosis of *rhodesiense* HAT in Zambia’s tsetse-infested Luangwa valley.

## Methods

### Study area

**Fig. 1:**
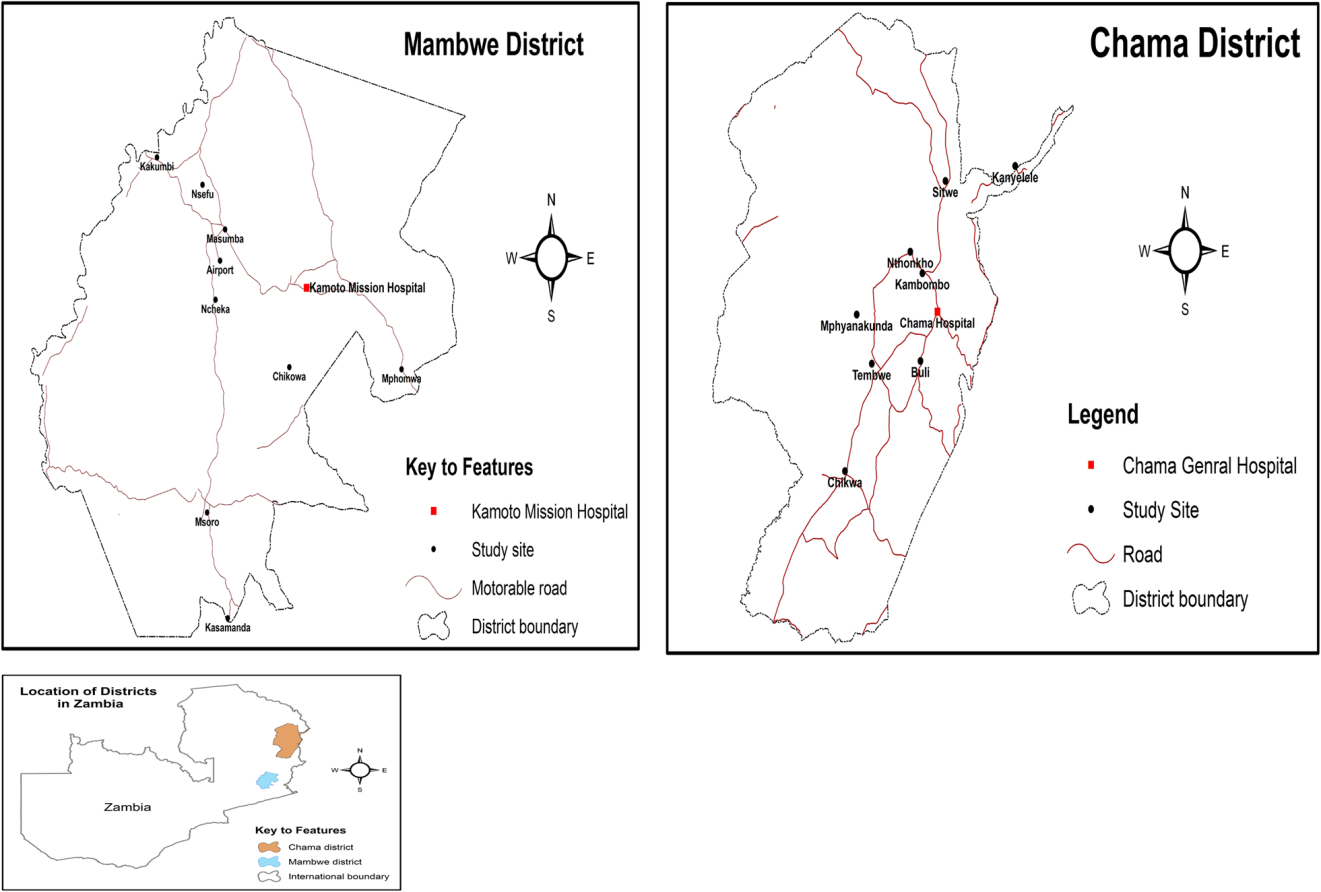
Map of Zambia showing location of the districts and health centres visited.* Source* CSO-Geographic Information Systems Unit

The study was conducted between April and July 2013 in Mambwe and Chama districts of the Eastern and Muchinga Provinces of Zambia, respectively (Fig. 1). The two districts were purposively selected because they are tsetse-infested, with abundant wild animals and HAT has previously been reported from them [4, 5, 9]. Mambwe district has a total area of 4840 square km with human population estimated at 68,918 while that of Chama district (with a surface area of 17,630 square km) is estimated at 103,894, making it the biggest district in eastern Zambia [21]. The total number of RHCs for Chama and Mambwe at the time the study was conducted was 20 and 13, respectively. In addition, Chama and Mambwe districts have a total of 136 and 77 health care personnel (HCP), respectively [22].

### Study design and data collection

The study was cross sectional. The number of cluster centres to be included in the study from each district was determined as previously described [23], giving 12 clusters for Chama and 9 for Mambwe districts, respectively. These centres were also selected purposively from the total RHCs in each district. However, due to low staffing levels of HCP at the health centres, the number of clusters was increased to 11 for Mambwe district. Thus the RHCs involved in the study included Tembwe, Kambombo, Nthonkho, Buli, Sitwe, Kalovia, Kanyelele, Mphyanakunda, Katangalika, Chama general, Chikwa and Kambwili (Chama district) and Kamoto mission, Chikowa, Airport, Ncheka, Chilanga, Masumba, Kakumbi, Nsefu, Mpomwa, St. Lukes in Msoro and Kasamanda (Mambwe district) (Fig. 1).

A structured questionnaire was administered to medical officers, clinical officers, nurses, environmental health technicians and/or laboratory technicians, who were present at the visited RHCs and interviewed on the availability of HAT diagnostic tools at their centres, their levels of HAT knowledge, their HAT awareness, how they diagnosed HAT and the number of such cases previously handled. For the purpose of this study, all these categories of staff were referred to as HCP and were included in the study. At least 50 % of the total number of the categorized HCP present during the visit were enrolled from each district. Thus, 52 and 49 HCP were enrolled from Chama and Mambwe districts, respectively.

### Data analysis

The data collected from the questionnaires were entered, stored and statistically analyzed using STATA version 11.0. The data from the interviews were summarized as frequencies and percentages and analyzed using descriptive statistics. Confidence interval was set at 95 %. The Chi square test was used to compare proportions between districts. For each analysis, Pearson’s Chi square P values <0.05 was considered statistically significant. Fisher’s exact test was used to compare proportions between districts where expected values or responses were less than five.

## Results

### Demographic data

A total of 101 HCP were captured in the study drawn from 23 RHCs in Chama and Mambwe districts. All the interviewed staff had attained secondary education while about 87 % (n = 101) of the HCP had continued their studies to tertiary level (Table 1). The two districts under study had similar staffing levels of various categories of HCP except for nurses who seemed to be more in Mambwe than Chama (Table 2). The overall number of the categorised staff at each particular RHC, however, was extremely low. In particular, both districts reported very low numbers of medical and clinical officers, who in most cases provide the first point of care to patients visiting a health facility. Only Chama general and Kamoto mission hospitals had doctors (two each).The duration of health service delivery in their particular districts for most of the staff involved in the study was short. It was observed that Mambwe district [59 % (29/49)] reported slightly more staff serving less than 5 years in the district compared to Chama district [48 % (25/52)] which reported relatively more staff serving over 5 years.

**Table 1:** Level of education of health care personnel involved in the study

District	University	College	Secondary only	Total
Chama	2 (3.8 %)	39 (75.0 %)	11 (21.2 %)	52
Mambwe	3 (6.1 %)	44 (89.8 %)	2 (4.1 %)	49
Total	5 (5.0 %)	83 (82.2 %)	13 (12. 9 %)	101

**Table 2:** Occupation of health care personnel involved in the study

District	Medical officers	Clinical officers	Nurses	Environmental health technicians	Laboratory technicians	Other^a^	Total
Chama	2 (3.8 %)	7 (3.5 %)	22 (42.3 %)	5 (9.6 %)	4 (7.7 %)	12 (23.0 %)	52
Mambwe	2 (4.1 %)	3 (6.1 %)	31 (63.3 %)	4 (8.1 %)	6 (12.2 %)	3 (6.1 %)	49
Total	4 (4.0 %)	10 (9.9 %)	53 (52.5 %)	9 (8.9 %)	10 (9.9 %)	15 (14.8 %)	101

^a^Community health assistants and professions not specified in the various staff categories

### Knowledge and awareness of HAT

We next investigated whether or not the HCP were able to suspect and identify a case of HAT in a patient. Malaria, diarrhoea, pneumonia and non-pneumonia respiratory tract infections were the four most common diseases reported by HCP from both Mambwe and Chama districts. Importantly, respondents from both districts had similar basic knowledge on how to identify a suspected case of HAT (Table 3). Most of the respondents indicated that HAT could be suspected upon a case of continuous intermittent fever and headache as well as abnormal sleep and non-responsiveness to anti-malarial drugs. In addition, the respondents submitted that microscopy provided definitive diagnosis of HAT in all the reported cases. The HCP from both districts indicated that they had previously encountered suspected HAT patients, with malaria-like symptoms but negative for malaria test based on laboratory evidence (mainly rapid diagnostic tests, RDTs) (χ^2^ = 2.17, df = 2, P > 0.05). Of note, whereas about 40 % of the HCP from Chama district (n = 52) indicated that they had encountered at least one case of HAT, only about 4 % of their Mambwe counterparts had similar experiences (n = 49) (χ^2^ = 18.90, df = 2, P = 0.000).

**Table 3:** Health care personnel’s knowledge of various signs and symptoms associated with HAT

Series	Parameter	Chama district (responses)		Mambwe district (responses)	
		Yes (%)	No (%)	Yes (%)	No (%)
1	Abnormal sleep	41 (78.8)	10 (21.2)	39 (79.6)	10 (20.4)
2	Fever	39 (75.0)	13 (25.0)	36 (73.5)	13 (26.5)
3	Body pains	25 (48.1)	27 (51.9)	21 (42.9)	28 (57.1)
4	Headache	27 (51.9)	25 (48.1)	22 (44.9)	27 (55.1)
5	Lymph node enlargement	13 (25.0)	39 (75.0)	11 (22.4)	38 (77.6)
6	Microscopy	26 (50.0)	26 (50.0)	27 (55.1)	22 (44.9)

### Availability of tools/equipment for HAT diagnosis and reported HAT cases

We next investigated if the RHCs had diagnostic tools for HAT. According to the obtained responses, the RHCs under study had the basic equipment and reagents for blood collection and detection of haemoparasites. Although a tendency towards having slightly more basic HAT diagnostic tools was observed in Mambwe district compared to Chama, this was not statistically significant. Importantly functional laboratories were only reported in Chama general and Kamoto mission hospitals which were normally used as referral hospitals for cases from RHCs. It was further observed that neither of the districts had PCR nor LAMP diagnostic tools.

It was also revealed that between 2003 and 2013, about 43 suspected HAT cases were reported from Chama district, of which 8 were confirmed by microscopy and treated. On the other hand, Mambwe district only reported one case (in March 2013) during the same period that was confirmed and later referred to the university teaching hospital in Lusaka for staging and treatment.

## Discussion

In Zambia, HAT is caused by *T. b. rhodesiense*, transmitted by tsetse species of *Glossina morsitans morsitans, Glossina pallidipes, Glossina morsitans centralis* and *Glossina brevipalpis*. Zambia has been reporting sporadic cases of the *rhodesiense* HAT, mainly in the old foci along the Luangwa river valley in north-eastern part of the country, including Chama, Mpika, Chipata and Mambwe districts [4, 5, 9]. In addition, sporadic *rhodesiense* HAT cases have recently been on the increase in Zambia’s Rufunsa district [unpublished reports]. Most of the HAT cases in Zambia are detected passively during routine visits to the health centres and, in several cases, when other haemoparasites (mainly malaria) are suspected and a blood smear is being examined. Unfortunately, most of these HAT cases, reported from very remote rural areas amongst the poorest of the poor where social amenities are either weak or non-existent [24], are either reported when the parasites have already crossed the patient’s blood–brain barriers (late stage HAT) as they are usually initially misdiagnosed for malaria, tuberculosis or HIV/AIDs, or in most unfortunate circumstances, patients may die without knowing the cause of their illness. It is possible that these cases could represent several others that may be unreported. According to Odiit et al. [14], about 39 % of *rhodesiense* HAT cases and 92 % of the deaths it causes, are unreported.

The present study, conducted through questionnaires and interviews, was an attempt to investigate the staffing levels, the HCP’s levels of alertness to the occurrence of HAT in Zambia’s tsetse-infested RHCs of Chama district as well as their capacity to detect such HAT patients, and make comparisons with the situation in Mambwe district. According to the obtained data, the levels of categorized staff at the RHCs were extremely low in both districts. Of note, only Chama general hospital and Kamoto mission hospital had medical doctors i.e. two doctors each, while only ten clinical officers were recorded among the 23 RHCs in the 2 districts. Most of the RHCs were manned by only one trained HCP, in most cases a nurse, sometimes assisted by ancillary staff such as community health assistants. The extremely low categorized HCP at RHCs is not surprising as the majority of the health personnel prefer to work in urban areas and are unwilling to work in rural areas due to poor road network and transportation, poor housing, limited facilities at RHCs and the general lack of social amenities. There is need for responsible authorities and the government to come up with deliberate policies that provide incentives to attract, motivate and retain health workers in RHCs. In such RHCs, the few available HCP have to attend to all the health problems of the local community and are further involved in clinical and laboratory diagnoses [5]. In this study, only Chama general and Kamoto mission hospitals had functional laboratories with qualified laboratory technicians. The two health facilities were also the only ones with doctors (two at each hospital) and hence offered points of referrals from the rural health centre network. As such, detection of endemic diseases including HAT may not be effectively done in most of the RHCs even when diagnostic tools are present.

In view of the fact that malaria, mainly detected by RDTs, is the major reported febrile disease in Zambia and was in the present study among the topmost four frequently reported diseases in the RHCs under study, most patients are treated with anti-malarial drugs on the basis of clinical diagnosis without laboratory confirmation. In such patients, HAT could easily be mistaken and treated as malaria due to the similarities in their clinical manifestations [10, 25]. There is also a possibility that HAT may co-exist with other endemic diseases including malaria, HIV/AIDS and tuberculosis, in which case the co-infected patients may be treated only for malaria but may apparently be resistant to the administered drugs [10–12]. In such HAT patients, multiple co-infections may influence the disease pathogenesis and complicate its management [12]. Even when a laboratory test is done, because RDTs are now more frequently used in most of the RHCs since they are more user-friendly and the fact that several RHCs do not have microscopes as reported in the present study, it is possible that HAT cases may easily be missed or misdiagnosed. Thus, although RDTs have been very helpful in diagnosing specific haemoparasites (mainly malaria parasites), their constant use have resulted in reduced diagnosis of other blood borne diseases which could normally be detected by microscopy. Taken together, these observations have serious implications in view of the fact that HAT requires specific treatment regimen during each of the disease phases [10] without which life could easily be lost.

Although the majority of the HCP in RHCs from both districts had attained tertiary education and their RHCs had basic diagnostic tools for HAT, it is possible that the extremely low staffing levels may have resulted in their inability to effectively and efficiently diagnose HAT and other endemic diseases [5, 13]. Failure to detect HAT cases contribute to the current challenges of HAT elimination in tsetse-infested regions [26]. However, the higher levels of awareness of HAT in Chama district could mainly be attributed to some HAT surveillance programs conducted between 2008 and 2012 during which period Chama was considered to be a HAT “hotspot” [4]. In 2008 alone, between the months of March and July, about 12 suspected HAT cases involving Zambia wildlife authority staff assigned to the then newly opened Mbambanda Zaro sanctuary, were reported in Chama district along the borders of Zambia and Malawi [27]. It must be emphasized that when a HAT case is diagnosed in the laboratory, there is a lot of publicity about it among HCP who usually encounter and attend to that case at one time or another during the long period when the patient is admitted for treatment. That could explain why close to half the number of HCP in Chama claimed they had previously encountered a HAT case. On the other hand, the very few sporadic cases being reported from Mambwe district may explain the scanty number of HCP who claimed to have had previous encounters with a HAT patient.

## Conclusions

This study has provided evidence on the various challenges HCP face in their quest to diagnose and manage HAT, among various endemic diseases in Zambia’s tsetse-infested Chama and Mambwe districts within the Luangwa river valley. The extremely low numbers of qualified HCP in the RHCs, the general absence of functional laboratories, coupled with absence of national HAT surveillance and control programs, should be addressed for effective management and control of the disease in such endemic regions.

## Abbreviations

AIDS: acquired immune deficiency syndrome
HAT: human African trypanosomiasis
HIV: human immune-deficiency virus
NTDs: neglected tropical diseases
PCR: polymerase chain reaction
RHC: rural health centre
SSA: sub-Saharan Africa
WHO: World Health Organization

## References

[1] Engels D, Savioli L (2006). Reconsidering the underestimated burden caused by neglected tropical diseases. Trends Parasitol.

[2] Namangala B, Oparaocha E, Kajino K, Hayashida K, Moonga L, Inoue N, Suzuki Y, Sugimoto C (2013). Preliminary investigation of trypanosomosis in exotic dog breeds from Zambia’s Luangwa and Zambezi valleys using LAMP. Am J Trop Med Hyg.

[3] Lisulo M, Sugimoto C, Kajino K, Hayashida K, Mudenda M, Moonga L, Nzala S, Namangala B (2014). Determination of the prevalence of African trypanosomiasis species in indigenous dogs of Mambwe district, eastern Zambia, by Loop-mediated isothermal amplification. Parasite Vectors.

[4] Namangala B, Hachaambwa L, Kajino K, Mweene AS, Hayashida K, Simuunza M, Simukoko H, Choongo K, Chansa P, Lakhi S, Moonga L, Chota A, Ndebe J, Nsakashalo-Senkwe M, Chizema E, Kasonka L, Sugimoto C (2012). The use of Loop-mediated Isothermal Amplification (LAMP) to detect the re-emerging Human African Trypanosomiasis (HAT) in the Luangwa and Zambezi valleys. Parasite Vectors.

[5] Mwanakasale V, Songolo P, Daka V (2013). Challenges in the control of Human African Trypanosomiasis in the Mpika district of Zambia. BMC Research Notes.

[6] Simukoko H, Marcotty T, Phiri I, Geysen D, Vercruysse J, Van den Bossche P (2007). The comparative role of cattle, goats and pigs in the epidemiology of livestock trypanosomiasis on the plateau of eastern Zambia. Vet Parasitol.

[7] Buyst H (1974). The epidemiology, clinical features, and treatment, and history of sleeping sickness in the Northern edge of Luangwa fly belt. Med J Zambia.

[8] World Health Organization (2015). Trypanosomiasis, human African (sleeping sickness).

[9] Mwanakasale V, Songolo P (2011). Disappearance of some human African trypanosomiasis transmission foci in Zambia in the absence of a tsetse fly and trypanosomiasis control program over a period of 40 years. Trans R Soc Trop Med Hyg.

[10] Legros D (2002). Treatment of human African trypanosomiasis-present situation and needs for research and development. Lancet Infect Dis.

[11] World Health Organization. Control and Surveillance of African Trypanosomiasis. Report of a WHO Expert Committee on Sleeping Sickness, Technical Report Series. Geneva, 1998. 10070249

[12] Kagira JM, Maina N, Njenga J, Karanja SM, Karori SM, Ngotho JM (2011). Prevalence and types of coinfections in sleeping sickness patients in kenya (2000/2009). J Trop Med.

[13] Chappuis F, Louis L, Simarro P, Lejon V, Buscher P (2005). Options for field diagnosis of human African trypanosomiasis. Clin Micro Rev.

[14] Odiit M, Coleman P, Liu W, McDermolt J, Fevre E, Welburn SC, Woolhouse MEJ (2005). Quantifying the level of under detection of *Trypanosoma brucei**rhodesiense* sleeping sickness cases. Trop Med Int Health.

[15] Njiru ZK (2012). Loop-mediated isothermal amplification technology: towards point of care diagnostics. PLoS Negl Trop Dis.

[16] Stich A, Abel PM, Krishna S (2002). Human African trypanosomiasis. BMJ.

[17] World Health Organization. Control of human African trypanosomiasis: a strategy for the African region. Geneva; 2005.

[18] Odiit M, Shaw A, Welburn SC, Fevre EM, Coleman PG, McDermott JJ (2004). Assessing the patterns of health-seeking behavior and awareness among sleeping sickness patients in eastern Uganda. Ann Trop Med Parasitol.

[19] Sindato C, Kimbita EN, Kibona SN (2008). Factors influencing individual and community participation in the control of tsetse flies and human African trypanosomiasis in Urambo District, Tanzania. Tanz J Health Res.

[20] John K, Kazwda R, Mfinanga G (2008). Knowledge of causes, clinical features and diagnosis of common zoonoses among medical practitioners in Tanzania. BMC Infect Dis.

[21] Central Statistics Office Zambia. Census of Population and Housing Central- Summary Report; 2010.

[22] Ministry of Health-Zambia: Staff management records. 2009.

[23] The role of SMEs in Rwanda from 1995 to 2010. http://www.memoireonline.com. Accessed on 07 Jun 2012.

[24] Simarro PP, Jannin J, Cottand P (2008). Eliminating human African trypanosomiasis: where do we stand and what comes next?. PLoS Med.

[25] Malele II, Kibona SN, Matemba LE, Sahani K, Swilla J, Mwalimu CD, Mayala BK, Kimaro E, Msumary C, Kalinga RB (2006). Human African Trypanosomiasis and challenges to its control in Urambo, Kasulu and Kibondo districts, western Tanzania. Tanz Health Res Bull.

[26] World Health Organization: Human African Trypanosomiasis (sleeping sickness): Epidemiological update. Geneva. 2006. 16673459

[27] Zambia wildlife authority report. Trypanosomiasis (Sleeping Sickness) infection among Bambanda-Zaro Sanctuary staff. 2008.

